# Phase 1 Dose-Escalation Study of Pegylated Arginine Deiminase, Cisplatin, and Pemetrexed in Patients With Argininosuccinate Synthetase 1–Deficient Thoracic Cancers

**DOI:** 10.1200/JCO.2016.71.3230

**Published:** 2017-04-07

**Authors:** Emma Beddowes, James Spicer, Pui Ying Chan, Ramsay Khadeir, Javier Garcia Corbacho, Dimitra Repana, Jeremy P. Steele, Peter Schmid, Teresa Szyszko, Gary Cook, Monica Diaz, Xiaoxing Feng, Amanda Johnston, Jim Thomson, Michael Sheaff, Bor-Wen Wu, John Bomalaski, Simon Pacey, Peter W. Szlosarek

**Affiliations:** Emma Beddowes, Javier Garcia Corbacho, and Simon Pacey, University of Cambridge, Cambridge; James Spicer, Dimitra Repana, Teresa Szyszko, and Gary Cook, King's College London; Pui Ying Chan, Jeremy P. Steele, Peter Schmid, Michael Sheaff, and Peter W. Szlosarek, St Bartholomew’s Hospital; Ramsay Khadeir, Peter Schmid, and Peter W. Szlosarek, Queen Mary University of London, London, United Kindgom; and Monica Diaz, Xiaoxing Feng, Amanda Johnston, Jim Thomson, Bor-Wen Wu, and John Bomalaski, Polaris Pharmaceuticals, San Diego, CA.

## Abstract

**Purpose:**

Pegylated arginine deiminase (ADI-PEG 20) depletes essential amino acid levels in argininosuccinate synthetase 1 (ASS1) –negative tumors by converting arginine to citrulline and ammonia. The main aim of this study was to determine the recommended dose, safety, and tolerability of ADI-PEG 20, cisplatin, and pemetrexed in patients with ASS1-deficient malignant pleural mesothelioma (MPM) or non–small-cell lung cancer (NSCLC).

**Patients and Methods:**

Using a 3 + 3 + 3 dose-escalation study, nine chemotherapy-naïve patients (five MPM, four NSCLC) received weekly ADI-PEG 20 doses of 18 mg/m^2^, 27 mg/m^2^, or 36 mg/m^2^, together with pemetrexed 500 mg/m^2^ and cisplatin 75 mg/m^2^ which were given every three weeks (maximum of six cycles). Patients achieving stable disease or better could continue ADI-PEG 20 monotherapy until disease progression or withdrawal. Adverse events were assessed by Common Terminology Criteria for Adverse Events version 4.03, and pharmacodynamics and immunogenicity were also evaluated. Tumor response was assessed by Response Evaluation Criteria in Solid Tumors (RECIST) version 1.1 for NSCLC and by modified RECIST criteria for MPM.

**Results:**

No dose-limiting toxicities were reported; nine of 38 reported adverse events (all grade 1 or 2) were related to ADI-PEG 20. Circulating arginine concentrations declined rapidly, and citrulline levels increased; both changes persisted at 18 weeks. Partial responses were observed in seven of nine patients (78%), including three with either sarcomatoid or biphasic MPM.

**Conclusion:**

Target engagement with depletion of arginine was maintained throughout treatment with no dose-limiting toxicities. In this biomarker-selected group of patients with ASS1-deficient cancers, clinical activity was observed in patients with poor-prognosis tumors. Therefore, we recommend a dose for future studies of weekly ADI-PEG 20 36 mg/m^2^ plus three-weekly cisplatin 75 mg/m^2^ and pemetrexed 500 mg/m^2^.

## INTRODUCTION

Standard-of-care first-line cytotoxic chemotherapy for many patients with advanced malignant pleural mesothelioma (MPM) and nonsquamous, non–small-cell lung cancer (NSCLC) is cisplatin plus pemetrexed. The overall prognosis for these patients remains poor despite treatment, and the majority of patients survive only 12 months.^1,2^ The development of novel treatment approaches is therefore of paramount importance for these patients.

Arginine is a semi-essential amino acid, which in normal cells can be synthesized de novo from citrulline combined with aspartate in the presence of ATP in the urea cycle, in addition to direct uptake of extracellular arginine. However, it has been found that a number of tumor types (eg, melanoma and prostate and ovarian cancers) have abnormalities in arginine synthesis pathways so that tumor cells are not able to synthesize arginine de novo and are dependent on an exogenous supply for growth (termed arginine auxotrophy).^3^ Arginine promotes tumor growth, and the observation that arginine-depleted feed is associated with reduced xenograft tumor growth was made in 1930.^4^ More recently, arginine deprivation studies using selected in vitro cancer cell lines have reported apoptosis of up to 80% of cells.^5^

A key enzyme in the biosynthesis of arginine is argininosuccinate synthetase 1 (ASS1), which combines citrulline with asparate to form argininosuccinate. Intratumoral deficiency of ASS1 has been detected in significant numbers of patients with cancers including mesothelioma and NSCLC.^6,7^ Epigenetic modification via aberrant methylation of the ASS1 promoter is proposed as underlying this deficiency, especially in mesothelioma.^7,8^ Importantly, low ASS1 expression is associated with a more aggressive clinical phenotype and a worse clinical outcome in several different cancer types.^9-12^ Moreover, it is known that patients resistant to the antifolate pemetrexed have high levels of thymidylate synthase and low levels of ASS1.^11^ Indeed, recent work indicates that low ASS1 promotes the diversion of aspartate for pyrimidine synthesis and enhanced tumor-cell proliferation.^13^ Thus, it is noteworthy that patients selected on the basis of low ASS1 expression are likely to be a cohort with worse prognosis and a poor response to currently used chemotherapy regimens.

Arginine deiminase catalyzes the conversion of arginine to citrulline, thereby depleting the former in ASS1-deficient tumor cells. Because the addition of polyethylene glycol to arginine deiminase increases bioavailability and decreases immunogenicity, pegylated arginine deiminase (ADI-PEG 20) was developed for clinical use.^14^ ADI-PEG 20 was well tolerated in early clinical trials as a single agent with promising biologic activity including for patients with hepatocellular carcinoma, metastatic melanoma, and mesothelioma.^15-20^ More recently, we showed, we believe for the first time, that single agent ADI-PEG 20 significantly extended progression-free survival (PFS) for patients with ASS1-deficient mesothelioma compared with best supportive care alone (Arginine Deiminase And Mesothelioma [ADAM] study).^8^

In preclinical studies, it was shown that the combination of arginine deprivation (using ADI-PEG 20) and pemetrexed leads to a potentiation of cytotoxicity in ASS1-negative tumor cells. We noted suppression of de novo thymidine synthesis with decreased levels of thymidylate synthase and inhibition of the salvage pathway via reduced thymidine kinase.^11^ Indeed, several pharmacologic and metabolic tracing studies support enhanced sensitivity to antifolate agents in arginine auxotrophs exposed to arginine-depleting agents.^13,21^ Furthermore, the addition of cisplatin to ADI-PEG 20 exerted, at the least, additive anticancer effects in both tissue culture and xenograft models of melanoma, and this mechanism is thought to be caused, at least in part, by the inhibition of DNA repair enzymes.^22^

We undertook a phase 1 dose-escalation study of ADI-PEG 20 combined with pemetrexed and cisplatin (ADIPemCis) in the first-line treatment of patients with nonsquamous NSCLC or mesothelioma tumors that were ASS1 deficient. The main aims of the study were to define the toxicity profile of the combination, to recommend phase 2 doses for ADIPemCis, and to investigate pharmacodynamic alterations in arginine metabolism.

## PATIENTS AND METHODS

### Study Design and Treatment

This was a multicenter, open-label, phase 1, dose-escalation trial designed to evaluate the safety and tolerability of ADI-PEG 20 combined with cisplatin and pemetrexed in patients with histologically proven advanced MPM and stage IIIB or IV nonsquamous NSCLC. A 3 + 3 + 3 phase 1 dose-escalation design was used to accommodate the predicted toxicity of pemetrexed and cisplatin.^1,23^

Preplanned dose levels of weekly intramuscular ADI-PEG 20 were 18 mg/m^2^, 27 mg/m^2^, and 36 mg/m^2^, together with intravenous treatment with cisplatin 75 mg/m^2^ and pemetrexed 500 mg/m^2^ every 3 weeks. The initial dose of ADI-PEG 20 was administered at least 48 hours before the first dose of cytotoxic drugs. To ameliorate toxicity from pemetrexed, patients received daily folic acid supplementation 400 μg and intramuscular hydroxycobalamin 1,000 μg every 9 weeks, both started at least 7 days before the first dose.

At least three patients were investigated at each dose level for a minimum of 21 days before escalation to the next cohort dose level. There was no intrapatient dose escalation. Patients continued ADIPemCis combination therapy for a maximum of six cycles (18 weeks). Patients with clinical benefit (stable disease or better) were eligible to receive continued single-agent ADI-PEG 20 treatment until disease progression.

The primary objectives of the trial were to evaluate the safety and tolerability of the combination (ADIPemCis) treatment and to establish a recommended phase 2 dose. Secondary objectives were to determine progression-free survival (PFS), overall survival (OS) at 1 year, and the pharmacodynamics of ADI-PEG 20 in combination with pemetrexed and cisplatin.

The study was performed in accordance with good clinical practice and the European Union Clinical Trials Directive, with approval from Leeds East (Yorkshire and The Humber) ethical review board. All patients provided written informed consent.

### Eligibility

Patients were 18 years of age or older, with histologically proven advanced MPM or stage IIIB or IV nonsquamous NSCLC. In addition, tumors were ASS1 deficient by immunohistochemistry, which was defined as a loss of ASS1 expression (0 or 1 plus immunohistochemistry staining)^7^ in > 50% of tumor cells. The 50% threshold was selected on the assumption that this would enrich the fraction of tumor cells likely to respond to arginine deprivation on the basis of multiple prior cell-line studies showing an inverse correlation between ASS1 expression and sensitivity to arginine depletion.^3,24^ Specifically, a statistically significant improvement in PFS for patients with mesothelioma was observed in the ADAM study using the 50% cutoff, with the hazard ratio decreasing further for tumors with > 75% ASS1 loss.^8^ Patients had evaluable disease by modified Response Evaluation Criteria in Solid Tumors (RECIST) for MPM and by RECIST 1.1 for nonsquamous NSCLC. All patients were chemotherapy naïve. Additional criteria included Eastern Cooperative Oncology Group performance status 0 or 1, adequate hematologic, hepatic, and renal function, and a minimum expected survival of 3 months. Exclusion criteria included anticancer therapy within 4 weeks of entering the study, ongoing toxic manifestations of previous treatments, symptomatic brain or spinal cord metastases, significant concomitant or uncontrolled intercurrent illness, recent major surgery, therapeutic anticoagulation, participation in another interventional clinical study, history of another primary cancer (unless treated curatively or unlikely to affect patient outcome), allergy to platinum salts or pegylated or *Escherichia* *coli* products, pregnancy, history of seizure disorder, and previous therapy with ADI-PEG 20.

### Safety Evaluations

Data on baseline characteristics of age, sex, performance status, and histology were collected for all patients. Physical examination was performed on day 1 of every cycle and at other times as clinically indicated. All treated patients were evaluated for safety by laboratory tests, physical examination, and adverse event (AE) assessment at screening, and at every 3 weeks during therapy. All AEs were graded according to the National Cancer Institute Common Terminology Criteria for Adverse Events version 4.03. AE monitoring continued for 30 days after the final treatment, and monitoring of AEs related to ADI-PEG 20 was continued until stabilization or resolution. Dose-limiting toxicities (DLTs) were assessed during cycle one (21 days) as AEs that were possibly, probably, or definitely related to study treatment, including grade 4 neutropenia (> 7 days duration); febrile neutropenia; grade 4 anemia (requiring transfusion therapy); grade 4 thrombocytopenia; or grade 3 or 4 nonhematologic toxicity with the exception of grade 3 nausea, vomiting, or diarrhea that resolved to a lower grade with supportive treatment within 7 days, grade 3 AST/ALT elevation without accompanying increase in bilirubin, alopecia, electrolyte abnormalities, or other grade 3 or 4 asymptomatic laboratory evaluations only deemed as DLTs if assessed clinically significant by the investigator; and a delay in cycle 2 for > 3 weeks. Patients were evaluable for DLTs if they had received at least 1 dose of ADI-PEG 20. Nonevaluable patients (those not receiving at least 1 dose of ADI-PEG 20) were replaced.

### Pharmacodynamic Evaluations

Blood samples were taken before each dose of ADI-PEG 20 to analyze arginine and citrulline levels and for immunogenicity analyses, as described previously.^8^

### Efficacy Evaluations

Computed tomography scans were performed every 6 weeks during ADIPemCis combination dosing and after every 8 weeks during ADI-PEG 20–only treatment. Tumor measurements were recorded and assessed according to RECIST 1.1 or modified RECIST.

## RESULTS

**Fig 1:**
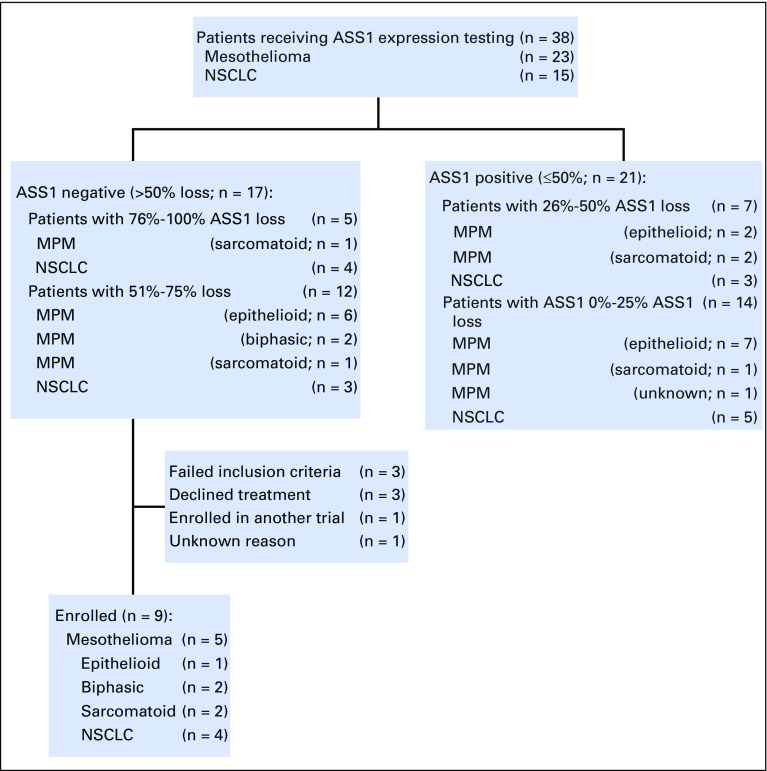
Patient screening and enrollment. ASS1, argininosuccinate synthetase 1; MPM, malignant pleural mesothelioma; NSCLC, non–small-cell lung cancer.

Nine eligible patients (four with NSCLC and five with MPM) were treated. Three patients were treated in each of the planned 18 mg/m^2^, 27 mg/m^2^, and 36 mg/m^2^ ADI-PEG 20 cohorts. A majority of patients managed to complete six cycles of combination treatment. The median number of weeks that the patients received treatment was 23.5 (range, 13 to 47 weeks) for patients with NSCLC and 31.0 (range, 30 to 47 weeks) for patients with MPM. Patients’ characteristics and dispositions are summarized in Table 1 and Figure 1.

**Table 1:**
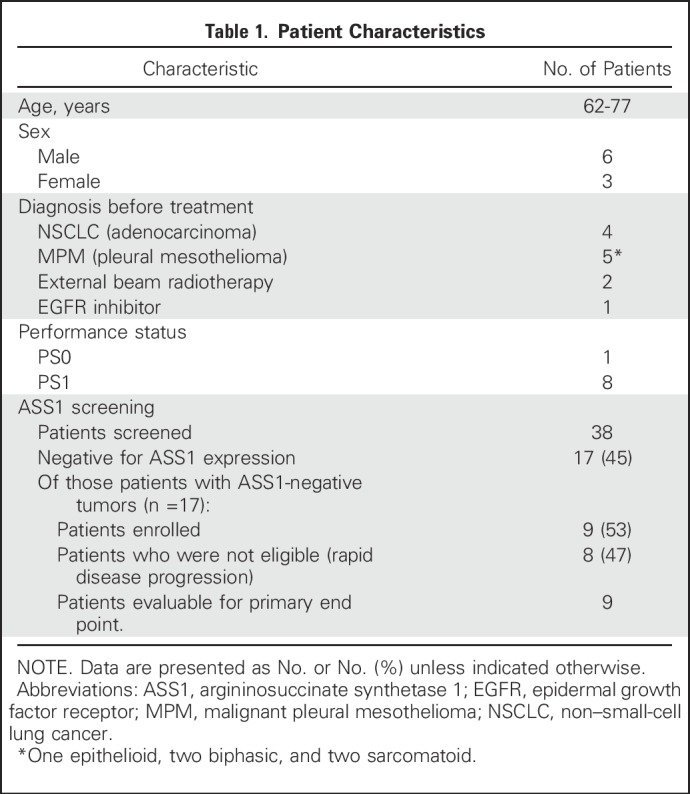
**Patient Characteristics**

### Safety

Most adverse events were grade 1 (83% of total) or grade 2. All AEs are summarized in Table 2. The most common toxicities were fatigue, nausea, vomiting, oropharyngeal toxicity (stomatitis, mucositis, and oral candidiasis), and rash. Hypersensitivity was observed in one patient and was attributed to cisplatin; this patient was rechallenged successfully with ADI-PEG 20. Febrile neutropenia was not observed; grade 3 neutropenia was reported in one of nine patients (11%). There were no DLTs or treatment-related deaths. Most AEs were attributed to (and expected as a result of) pemetrexed and/or cisplatin therapy; only nine of 38 (grade 1 or 2) reported AEs were related to ADI-PEG 20. In this small number of patients, there was no clear dose–response relationship between ADI-PEG 20 dose level and toxicity.

**Table 2:**
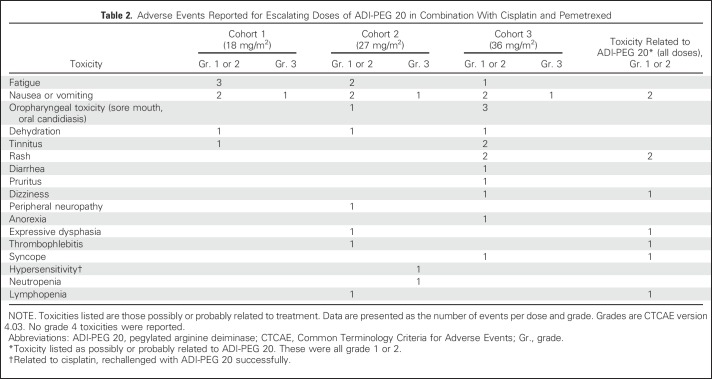
Adverse Events Reported for Escalating Doses of ADI-PEG 20 in Combination With Cisplatin and Pemetrexed

### Pharmacodynamics

**Fig 2:**
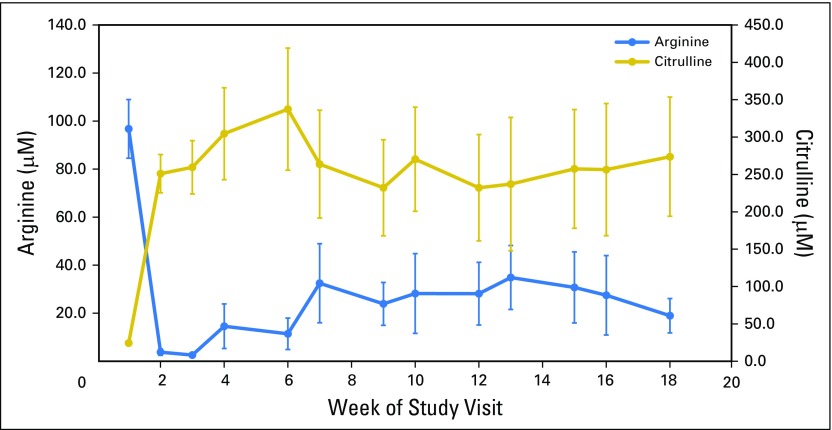
Pharmacodynamics of arginine and citrulline in patients treated with pegylated arginine deiminase combined with pemetrexed and cisplatin. Median serum concentrations of both arginine and citrulline are shown by week of treatment. Error bars shown are ±SEM.

**Fig 3:**
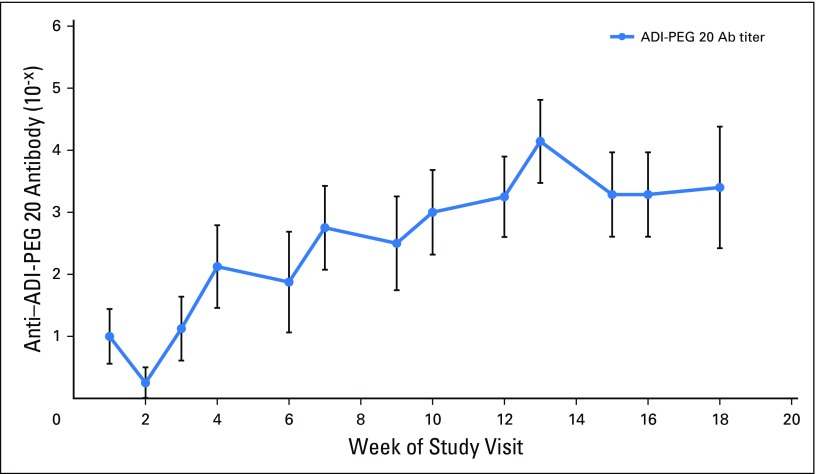
Mean serum levels of anti–ADI-PEG 20 antibodies in all patients by week of treatment with ADI-PEG 20 combined with pemetrexed and cisplatin. Error bars shown are ± SEM. Ab, antibody, ADI-PEG 20, pegylated arginine deiminase.

Despite variability because of the small sample size, circulating plasma arginine concentrations were depleted rapidly in all patients and remained suppressed at approximately 30% of baseline levels throughout the 18 weeks of triplet therapy. Correspondingly, plasma levels of the ADI-PEG20 product citrulline increased rapidly and remained elevated during the same dosing period (Fig 2). Anti–ADI-PEG 20 antibody titers are illustrated in Figure 3. In summary, titers of antidrug antibodies increased gradually, seemed to plateau at week 8 to week 10, and remained below 10^−4^ by week 18.

### Response

**Fig 4:**
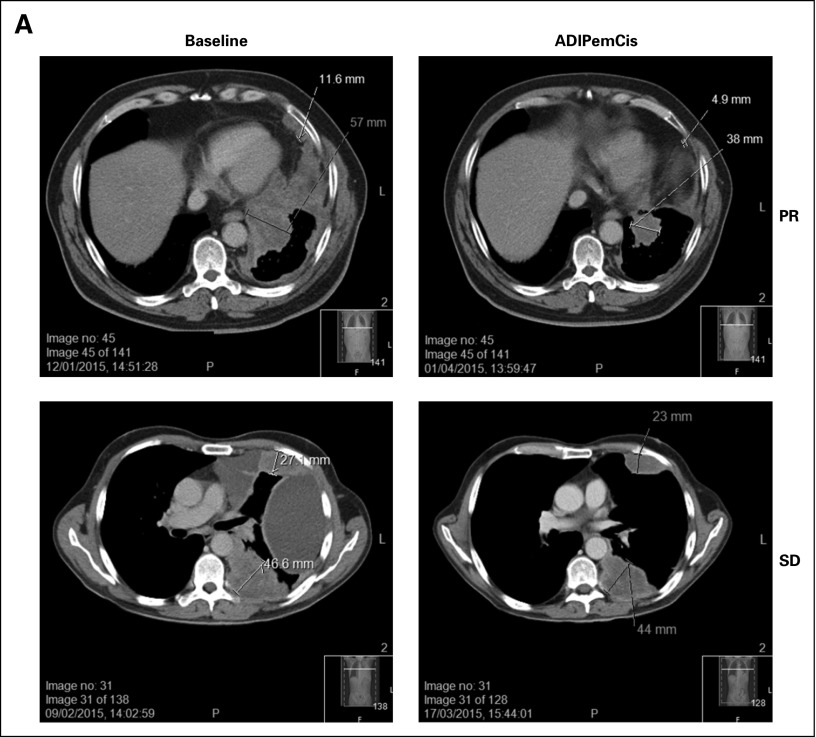

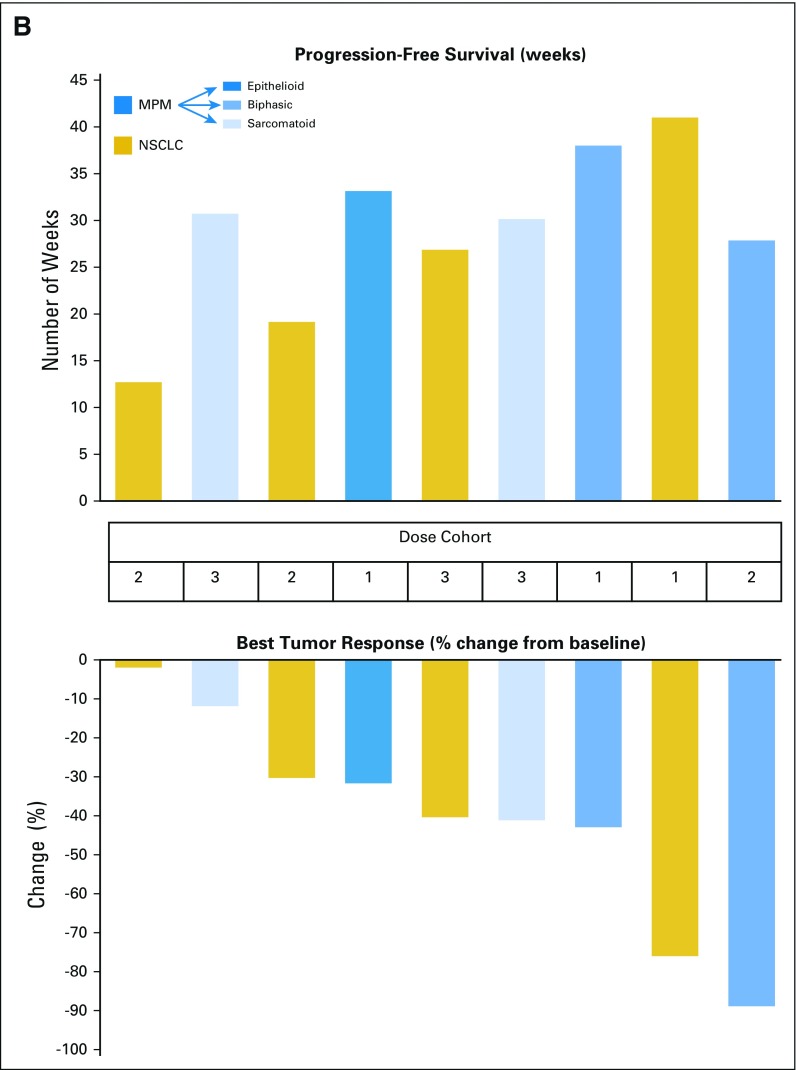
(A) Sarcomatoid MPM displaying PR and SD responses (cohort 3). (B) Progression-free survival shown for ADIPemCis together with corresponding best tumor response (percentage change compared with baseline) for all patients. Dose cohort of each patient is indicated (1 to 3). ADIPemCis, pegylated arginine deiminase combined with pemetrexed and cisplatin; MPM, malignant pleural mesothelioma; NSCLC, non–small-cell lung cancer; PR, partial response; SD, stable disease (and resolution of pleural effusion).

All patients experienced a best response of stable disease or better. Furthermore, seven of nine patients (78%) achieved a partial response, which was seen at all dose levels investigated (ie, overall response rate of 0.78; 95% CI, 0.39 to 0.97). The median OS was 55.7 weeks, and the median PFS was 30.1 weeks. The median OS was 56.4 weeks (range, 30.7 to ≥ 105.1 weeks), and the median PFS was 30.7 weeks (range, 27.9 to 38.0 weeks) for patients with MPM, whereas for patients with NSCLC, the median OS was 55.5 weeks (range, 25.7 to 56.7 weeks) and the median PFS was 23.0 weeks (range, 12.7 to 41.0 weeks). Individual patient data are summarized in Figure 4.

## DISCUSSION

In this biomarker-directed study, we have presented data on the first combination of the arginine-depleting agent (ADI-PEG 20) with cisplatin and pemetrexed (ADIPemCis). Patients with ASS1-deficient thoracic tumors were treated for up to six cycles, and treatment was well tolerated overall. The most common adverse effects were nausea and vomiting at a frequency comparable to that seen in previous studies of cisplatin and pemetrexed. The addition of ADI-PEG 20 prompted an increase in rash as the most common adverse effect. One hypersensitivity reaction occurred during the administration of cisplatin. This patient was subsequently rechallenged successfully with ADI-PEG 20. All the toxicities that were reported as being possibly or probably related to ADI-PEG 20 were grade 1 or 2 in nature. No DLTs were reported.

Arginine suppression with the ADIPemCis triplet was more prolonged than that which was observed previously with ADI-PEG 20 monotherapy. In the ADAM monotherapy study, arginine was depleted to below 30% of baseline for 7 weeks, but by 9 weeks, arginine concentrations had returned to pretreatment levels, with a corresponding fall in citrulline concentrations.^8^ Arginine suppression was also detected for the first 8 weeks only, before reaching baseline levels by week 12, in a recent phase 1 combination study of ADI-PEG20 combined with docetaxel in solid tumors.^25^ In contrast, with the ADIPemCis regimen, arginine concentrations remained depleted compared with baseline levels until the end of treatment, and citrulline concentrations were persistently elevated. Anti–ADI-PEG 20 antibodies increased gradually, then seemed to reach a plateau at approximately 10^−3^ by 8 to 10 weeks. Interestingly, this was in contrast to samples taken from patients treated in the monotherapy and docetaxel combination study, in which anti–ADI-PEG 20 antibodies increased rapidly and reached 10^−3^ by 9 weeks, followed by a continued increase in titers (10^−5^ to 10^−6^) before reaching a plateau by 16 weeks. Prolonged arginine depletion may be caused by the suppression of neutralizing anti–ADI-PEG 20 antibodies by pemetrexed and cisplatin, especially cisplatin, and by the use of concomitant corticosteroids as chemotherapy premedication.^26^

We report an interesting clinical activity signal in this small patient cohort, with an overall RECIST response rate of 78%. Previous studies reported response rates of 31% and 41% for cisplatin and pemetrexed, respectively, administered to patients with NSCLC and MPM.^1,2^ Importantly, two patients with biphasic MPM and one with sarcomatoid MPM treated with ADIPemCis in this study have achieved partial response. Sarcomatoid histology is considered resistant to chemotherapy, and with the combination of pemetrexed, cisplatin, and bevacizumab, there were no responses reported among the five patients with sarcomatoid MPM.^27^ A recent epidemiologic study reported a median OS of 13.3 versus 6.2 months for patients with epithelioid and nonepithelioid MPM, respectively.^28^ Recently, Bueno et al reported that the prognosis of patients with the non-epithelioid subtypes (sarcomatoid, biphasic-sarcomatoid, biphasic-epithelioid) was worse than that of patients with the pure epithelioid subtype of mesothelioma (*P* = .0001). Interestingly, this outcome correlated with significant downregulation of the ASS1 gene in sarcomatoid compared with epithelioid MPM.^29^ One of our patients with biphasic MPM remains alive 26 months after presentation, with a debilitating corticosteroid-refractory anticyclic citrullinated peptide antibody-positive paraneoplastic arthritis of the hands, described previously with several cancers but not with MPM.^30^ The arthritis resolved within the first cycle of ADIPemCis and then fluctuated at a lower intensity while the patient was receiving ADI-PEG20 monotherapy.

The median PFS and OS outcomes for the study overall are within the range expected for platinum and pemetrexed doublets but with less aggressive cancers. The clinical activity signal described, in particular in patients with MPM, is being studied further in an expansion cohort including ^18^F-fluorothymidine positron emission tomography scanning and will be reported separately.^31^

Several other triplet combination phase I trials with ADI-PEG 20 are ongoing, including one examining the combination of ADI-PEG 20 with gemcitabine and nab-paclitaxel in pancreatic cancer (ClinicalTrials.gov identifier NCT02101580) and another examining ADI-PEG 20 with FOLFOX in the treatment of advanced GI malignancies, especially hepatocellular carcinoma (ClinicalTrials.gov identifier CT02102022). The key rationale is similar in that significant proportions of these tumors are auxotrophic for arginine and in that the disruption of arginine supply with ADI-PEG 20 suppresses, in particular, nucleotide metabolism.^13,32,33^ These trials seek to emulate the success seen with asparaginase combination therapy in the treatment of acute lymphoblastic leukemia many years earlier, where a signal was observed with monotherapy but a significant number of cures were not seen until it was combined with multiple other agents.^34^ Importantly, the effects of combination chemotherapy are likely to be tumor cell type specific, as evidenced by the differential modulation of enzymes involved in nucleotide synthesis after treatment with ADI-PEG20 in mesothelioma compared with melanoma.^11,35^ In the case of platinum agents, resistance has been observed in ovarian cancer and NSCLC cell lines displaying loss of ASS1, whereas ADI-PEG 20 has been shown to induce cisplatin sensitivity in the latter.^9,36^ Further work will be needed to optimize antimetabolite combinations on the basis of careful biomarker analyses to identify the role of arginine deprivation in the clinic.^24^

In summary, our results are consistent with preclinical data that support a synergistic interaction of platinum-based and antifolate chemotherapy with concomitant arginine depletion in patients selected for tumors deficient in ASS1. The triplet regimen has achieved high response rates in this small trial, and was well tolerated in patients with MPM and NSCLC. With limited treatments available for these patients, this triplet combination warrants further study. The recommended phase 2 dose was weekly, intramuscular ADI-PEG 20 36 mg/m^2^ plus three-weekly intravenous cisplatin 75 mg/m^2^ and pemetrexed 500 mg/m^2^. A randomized phase 2–3 trial for ASS1-deficient patients with MPM has opened (ClinicalTrials.gov identifier NCT02709512).
